# Tumor-Suppressive Role of microRNA-202-3p in Hepatocellular Carcinoma Through the KDM3A/HOXA1/MEIS3 Pathway

**DOI:** 10.3389/fcell.2020.556004

**Published:** 2021-01-15

**Authors:** Yijie Zhang, Qi Pan, Zigong Shao

**Affiliations:** Department of Organ Transplantation and Hepatobiliary, Key Laboratory of Organ Transplantation of Liaoning Province, The First Affiliated Hospital, China Medical University, Shenyang, China

**Keywords:** hepatocellular carcinoma, microRNA-202-3p, KDM3A, HOXA1, MEIS3

## Abstract

Hepatocellular carcinoma (HCC) represents a malignant tumor predominantly arising in the setting of cirrhosis and is the third most common cause of cancer-associated death on a global scale. The heterogeneous nature of HCC and limited well-recognized biomarkers may contribute to poor patient prognosis and treatment failure. In this study, we identified expression pattern of microRNA-202-3p (miR-202-3p) in HCC and characterized its functional role as well as related mechanisms. First, we collected 50 HCC tissues and 38 normal liver tissues, and after bioinformatics prediction, the expression of miR-202-3p and KDM3A was determined in the tissues. We found lowly expressed miR-202-3p and overexpressed KDM3A in HCC tissues. Then, dual-luciferase reporter gene assay was employed to test the presence of miR-202-3p binding sites in the 3’UTR of KDM3A and chromatin immunoprecipitation (ChIP) assay to homeobox A1 (HOXA1) interaction with KDM3A and MEIS3. It has been confirmed that miR-202-3p negatively regulated KDM3A responsible for increasing the expression of HOXA1 by eliminating the histone H3 lysine 9 (H3K9)me2 in HCC cells. HOXA1 could evidently increase H3K4me1 and H3K27ac enrichment in the MEIS3 enhancer region and enhance the expression of MEIS3. Functional assays were also performed with the results showing that upregulated miR-202-3p or downregulated KDM3A retarded HCC cell viability, migration, and invasion. In addition, HepG2 cells were xenografted into nude mice, and we demonstrated that upregulated miR-202-3p reduced the growth of human HCC cells *in vivo*. Taken together, the present study elicits a novel miR-202-3p/KDM3A/HOXA1/MEIS3 pathway in HCC, potentiating an exquisite therapeutic target for HCC.

## Introduction

Hepatocellular carcinoma is the sixth most prevalent malignancy and ranks third in overall global cancer-associated mortality worldwide, accounting for an estimated one million causalities across the globe in 2030 (Villanueva, 2019). The mortality rate of HCC is only second to lung cancer in males due to gender stratification on the basis of the Global Cancer Statistics 2018 (Bray et al., 2018). Due to the late diagnoses of HCC in an advanced stage in most patients, it is associated with poor patient survival and treatment failure (Kulik and El-Serag, 2019). A comprehensive understanding of the signaling events that initiate or inhibit liver carcinogenesis is required to achieve early diagnosis and to further develop more effective therapeutic modalities.

Recent investigations have also highlighted the implications of microRNAs (miRNAs) in HCC due to their aberrant expression profile in HCC tissues and their regulatory roles in cell malignant behaviors (Forner, 2015). miRNAs, with lengths ranging from 18 to 24 nucleotides, have been underlined to function as either tumor suppressors or oncogenes by binding to their respective target messenger RNAs in mediation of cancer progression, drug resistance, metastasis, or relapse (Biswas, 2018). One particular study demonstrated the ability of miR-202 suppress HCC cell proliferation by post-transcriptionally inhibiting LRP6 from previous evidence (Zhang et al., 2014). Online prediction highlighted the presence of putative miR-202-3p binding sites in the 3′UTR of KDM3A. KDM3A, a H3K9 demethylase, is known to play an oncogenic role in human cancers including HCC (Nakatsuka et al., 2017). KDM3A has exhibited ability to transcriptionally increase the expression of HOXA1 by erasing the H3K9me2, thus promoting the cell cycle progression in cancer cells (Cho et al., 2012). In addition, *hepatitis C virus*, a well-recognized risk factor of HCC, possesses the ability to inhibit the transcription of miR-181c and thus increase the expression of HOXA1 (Mukherjee et al., 2014). Previous evidence shows HOXA1 are enriched in the enhancer region of MEIS3 (De Kumar et al., 2017). MEIS3 directly targets 3-phosphoinositide-dependent PDK1, a recognized kinase involved in the PI3K/Akt signaling pathway, and regulates cell survival (Liu et al., 2010). In light of these findings, we aimed to elucidate the functional roles of miR-202, KDM3A, HOXA1, and MEIS3 in HCC and their interactions. Our results provided new mechanistic insights for a better understanding of hepatotumorigenesis and revealed the functionality of the miR-202-3p/KDM3A/HOXA1/MEIS3 pathway as a potential therapeutic target for HCC.

## Materials and Methods

### Ethics Statement

The current study protocol was approved by the Ethics Committee of the First Affiliated Hospital of China Medical University (No. 20160105). Signed informed consent was obtained prior to subject enrollment. Animal studies were performed with the approval from the Institutional Animal Care and Use Committee of the First Affiliated Hospital of China Medical University (No. 20170111).

### Bioinformatics Analysis

Databases including TargetScan (Representative miRNA < −0.25)^1^, mirDIP (Integrated Score > 0.3)^2^, miRDB (Target Score > 80)^3^, DIANA TOOLS (miTG score > 0.5)^4^, RAID (Score > 0.6)^5^, and miRWalk (bindingp = 1, energy < -20, accessibility < 0.01, au > 0.5)^6^ was applied to predict the downstream genes of miR-202 separately. Then, Venn diagrams were drawn to obtain important downstream genes of miR-202, and Multiple Proteins function of String (minimum required interaction score = 0.150)^7^ was employed to construct a PPI network of important downstream genes. Cytoscape (Fig.^8^) was applied to beautify the picture and calculate the gene core degree to select the key genes with the highest core degree for further study. Afterward, the R language was used to obtain the expression trends of key genes in microarray GSE62232 from the GEO database^9^, and the downstream genes that may be regulated by key genes were identified by the available literature. By the Scan function of JASPAR^10^, the binding sites of key genes to their downstream genes were acquired, and the Pearson correlation of the expression of these two genes was analyzed through Pan-Cancer function of starBase^11^. Through the online analysis tool Multi Experiment Matrix (MEM)^12^, RRA (RobustRankAggreg) algorithm analysis was used to determine the degree of co-expression significance between genes, and the Select collection: A-AFFY-44 Affymetrix GeneChip Human Genome U133 Plus 2.0[HG-U133_Plus_2] and Distance measure: Pearson were selected in MEM co-expression analysis. Subsequently, the regulatory genes of downstream genes were further screened by existing studies. The expression trends of the regulatory genes were obtained through TCGA analysis module analysis of Ualcan^13^, and the expression correlations and risk profiles were given from starBase, and their co-expression relationships were determined by MEM.

### Human Tissue Specimens

The current study was based on 50 cancerous liver tissue specimens harvested from HCC patients (31 males and 19 females) who underwent first hepatectomy at the First Affiliated Hospital of China Medical University between February of 2016 and August of 2018, with resection of 38 normal liver tissue specimens from non-HCC patients as control. No patients had received radiotherapy, chemotherapy, or immunotherapy prior to the hepatectomy. The 50 enrolled patients aged from 26 to 78 years, with 33 cases over 50 years and 17 cases less than 50 years. Among the 50 HCC patients, 23 were classified as grade I-II and 27 were grade III-IV based on Edmondson-Steiner’s grading, and 21 were in stage I–II and 29 were in stage III–IV by tumor node metastasis (TNM) staging.

### Cell Lines

Four human hepatoma cell lines SMMC7721, HepG2, Hep3B, Huh7, and two normal human liver cell line cell lines LO2 and QSG-7701, all provided by the Institute of Basic Medical Sciences, Chinese Academy of Medical Sciences, were cultivated in the DMEM (Hyclone, Laboratories, Inc., Logan, UT, United States) with exposure to an atmosphere of 5% CO_2_ at 37°C. The medium comprised of 10% FBS (Sigma), 300 μg/mL L-glutamine, 400 U/mL penicillin, and 50 μg/mL streptomycin.

### Lentiviral Transduction and Cell Transfection

HepG2 cell suspension (2 mL, 2.5 × 10^4^ cells/mL) was seeded into a six-well plate and then added with HOXA1 or MEIS3 recombinant lentiviral expression vectors (2 × 10^6^ TU) pre-cultured in 1 mL serum-free and antibacterial medium and 5 μg Poly-brene. Forty-eight hours later, the HepG2 cells with stable HOXA1 or MEIS3 overexpression in the presence of puromycin we selected as the cell line for subsequent experimentation. Then, the lentivirus-infected HepG2 cells were treated with 100 nmol/L miR-202-3p mimic, or siRNA oligonucleotide duplexes (SIGMA Genosys) targeting the human KDM3A and HOXA1 transcripts for 48 h using the lipofectamine 2000 reagent (Invitrogen, United States) following standard protocols. siRNA targeting EGFP, siRNA targeting FFLuc, and scramble siRNA was used for negative control. Oligonucleotide duplexes of siRNA are listed in Table 1.

**TABLE 1:** A series of siRNA oligonucleotides.

siRNA	Oligonucleotides
Scramble siRNA	Sense: 5′-GCAGCACGACUUCUUCAAG-3′
	Antisense: 5′-CUUGAAGAAGUCGUGCUGC-3′
FFLuc-specific siRNA	Sense: 5′-GUGCGCUGCUGGUGCCAAC-3′
	Antisense: 5′-GUUGGCACCAGCAGCGCAC-3′
KDM3A-specific siRNA	Sense: 5′-GUCUAUGUGGGAAUUCCCA-3′
	Antisense: 5′-UGGGAAUUCCCACAUAGAC-3′
HOXA1-specific siRNA	Sense: 5′-GUUCCUUUCAGAUGACCUU-3′
	Antisense: 5′-AAGGUCAUCUGAAAGGAAC-3′

### Cell Viability Assay

Cell viability was examined by means of the MTT assay. In brief, HCC cells were cultured with 200 μL of the medium (4 × 10^3^ cells/well) and then preserved with 20 μL of 5 mg/mL MTT solution in a 96-well plate for 4 h. Two hours later, the medium in each well was renewed using 150 μL of DMSO (Sigma–Aldrich) for dissolution of the formazan crystals. Absorbance was evaluated at an excitation wavelength of 450 nm using a Microplate reader (US 6111636, Thermo Labsystems, Helsinki, Finland) at 24th, 48th, 72nd, and 96th h, with the growth curves plotted.

### Transwell Migration and Matrigel-Based Invasion Assays

Transwell chamber systems (Merck Millipore, Germany) were adopted for cell migration and invasion assays. In brief, the HCC cells were subjected to 2-h serum-free starvation and then resuspended using the serum-free medium, after which 200 μL of cell suspension (5 × 10^4^ cells) were added into the upper chambers. The lower chambers were supplemented with 10% FBS-supplemented DMEM (500 μL, BD Biosciences, United States). Following 24-h incubation at 37°C, both chambers were subjected to 4% paraformaldehyde fixation and 0.1% crystal violet staining. Stained cells in the lower chamber were counted in 10–20 random fields per well using the microscope. Cell migration and invasion assays shared same protocols except for the presence of 50 μL Matrigel in the upper chambers (Sigma–Aldrich, United States) for invasion assays.

### Western Blot Analysis

Tissue homogenate and cells were lyzed in ice-cold RIPA buffer supplemented with the protease inhibitor PMSF (1 mM). Following the protein quality control, equal amounts of protein samples (50 μg) underwent 10% SDS-PAGE (Bio-Rad, Hercules, CA, United States) and then transferred onto the nitrate membrane (Bio-Rad). Western blots were incubated with primary antibodies (Abcam, Cambridge, United Kingdom) and incubated with horseradish peroxidase-labeled goat anti-rabbit IgG (ab205718, 1:10,000). Each sample was repeated in triplicate. β-actin was used as a loading control for normalization. Next, Western blots were visualized using the enhanced ECL method as per the manufacturer’s protocol (BB-3501, Ameshame, United Kingdom) and analyzed using BIO-RAD gel image analysis system and Quantity One v4.6.2. Primary antibodies were as follows: rabbit anti-KDM3A (ab91252), rabbit anti-HOXA1 (ab37563), rabbit anti-MEIS3 (ab185961), and rabbit anti-β-actin (ab8226).

### RNA Extraction and Quantitative Real-Time PCR (qRT-PCR) Analysis

Total RNA was extracted from tissues and cells using the miRNeasy Mini Kit (QIAGEN, Germany). For miRNA, cDNA was generated using the miRNA First Strand cDNA Synthesis (Tailing Reaction) kit (B532451-0020, Shanghai Sangon, China) in strict accordance with the provided instructions (HaiGene, Harbin, China). For the mRNA, synthesis of cDNA was carried out using the kit (RR047A, Takara, Japan) following the instructions provided by the manufacturer. qRT-PCR was performed with the SYBR^®^Premix ExTaqTM II (RR820A, Takara) on the ABI PRISM^®^7300 System (Applied Biosystems, United States), with each reaction run in triplicate. Universal RT primer of miRNA and forward primer of U6 were provided by the miRNA First Strand cDNA Synthesis kit. The mRNA primer information is listed in Table 2. The miR-202-3p level was normalized to U6 and the other genes to GAPDH, respectively. Results were calculated based on the 2^–ΔΔ^^*CT*^ method.

**TABLE 2:** Primer sequences for RT-qPCR and ChIP analyses.

Target	Primer sequence
GAPDH-f	5′-GCAAATTCCATGGCACCGTC-3′
GAPDH-r	5′-TCGCCCCACTTGATTTTGG-3′
SDH-f	5′-TGGGAACAAGAGGGCATCTG-3′
SDH-r	5′-CCACCACTGCATCAAATTCATG-3′
KDM3A-f1	5′-CAGCCAGCACATCTCCTCTAAAC-3′
KDM3A-r1	5′-TGGATTTGCTTAAAGGTGGGAGG-3′
KDM3A-f2	5′-TCTCCTCTAAACTGGCTGGCCGAC-3′
KDM3A-r2	5′-TGTTAAACGTATGGAGGACTGTGC-3′
HOXA1-f	5′-CGGAACTGGAGAAGGAGTTC-3′
HOXA1-r	5′-TTCACTTGGGTCTCGTTGAG-3′
MEIS3-f	5′-TTCCTTCAACGAGGACATCGC-3′
MEIS3-r	5′-ATCTTTCCCTTCTCCAGCTCCA-3′
HOXA1-ChIP-f	5′-GCCCTCTTCCCTTCTCACCT-3′
HOXA1-ChIP-r	5′-GGCCTCCGGGAGGTGGGGGC-3′
MEIS3-ChIP-f	5′-CACTGTAAGTTATTGCCTCAAAGG-3′
MEIS3-ChIP-r	5′-AGCTTGTAATACTTGTGGGCTTT-3′

### Dual-Luciferase Reporter Gene Assay

The untranslated region (UTR) at the 3’ end of KDM3A-wild type (KDM3A-WT) containing the putative miR-202-3p binding sites and KDM3A mutated in the putative miR-202-3p binding sites (KDM3A-MUT) or HOXA1-WT were inserted into the pGL3-control luciferase vectors (Promega, United States), respectively. Next, the desired luciferase vectors with either miR-202-3p mimic or mimic NC were co-transfected into HEK293T cells. The luminescence of firefly luciferase was determined using dual-luciferase reporter assay system kit (Promega, United States) on the Luminometer TD-20/20, relative to that of renilla luciferase. The sequences of miR-202-3p mimic and mimic-NC are given in Table 3.

**TABLE 3:** Gene sequences.

Gene	Sequences
MiR-202-3p mimic	F: 5′-AGAGGTATAGGGCATGGGAA-3′
	R: 5′-TTCCCATGCCCTATACCTCT-3′
Mimic-NC	F: 5′-TTCTCCGAACGTGTCAGCTAA-3′
	R: 5′-ACGTGACACGTTCGGAGAATT-3′

*Note: F, forward; R, reverse.*

### Chromatin Immunoprecipitation (ChIP) Assay

Chromatin immunoprecipitation assays were performed using a commercially available kit (Millipore, Billerica, MA, United States) following the instructions provided by the manufacturer. In brief, cells were transfected with pCAGGS-n3FC (Mock), pCAGGSn3FC-KDM3A (FLAG-KDM3A), or pCAGGSn3FC-HOXA1 (FLAG-HOXA1) vectors, and KDM3A- or HOXA1-specific siRNA for 48 h, respectively. The fragment of KDM3A or HOXA1 and chromatin complexes was immnoprecipitated with anti-FLAG (M2, Sigma–Aldrich), anti-H3K9me2 (ab1220, Abcam), anti-H3K9me3 (ab8898, Abcam), anti-H3K4me1 (ab8895, Abcam), anti-H3K4me3 (ab8580, Abcam), anti-H3K27ac (ab4729, Abcam), and anti-H3K27me3 (ab6002, Abcam) antibodies. After elution of the bound DNA fragments, the amount was subjected to qRT-PCR analysis, with primer sequences shown in Table 2.

### Tumorigenicity Assays of Human HCC Cells in Nude Mice

A total of 24 SPF-conditioned nude mice (*n* = 24, aged 4–6 weeks, weighing 17–20 g) were subcutaneously injected with 200 μL miR-202-3p agomir and cell suspension prepared by incorporating 5 × 10^6^ HepG2 cells treated with HOXA1 recombinant lentiviral expression vectors and Matrigel at a dilution ratio of 1:1. A total of four mouse groups were detailed as follows: control (PBS injection only), agomir-NC + oe-NC, miR-202-3p agomir + oe-NC, and miR-202-3p agomir + oe-HOXA1. Tumor growth was examined every 3 days in total 30 days. All mice were euthanized by means of CO_2_ asphyxiation.

### Statistical Analysis

All data (mean ± standard deviation) were representative of three independent experiments (each in triplicate). Independent-sample *t*-test, a one-way ANOVA with Tukey’s test, and repeated measurements ANOVA with Bonferroni test were performed for appropriate statistical comparisons. All statistical analyses were performed using the SPSS 19.0 software (IBM, Armonk, NY, United States), with two-tailed value of *p* < 0.05 representative of a level of statistical significance.

## Results

### miR-202-3p Targeted KDM3A Gene and Inhibited HCC Cell Viability, Migration, and Invasion

**FIGURE 1:**
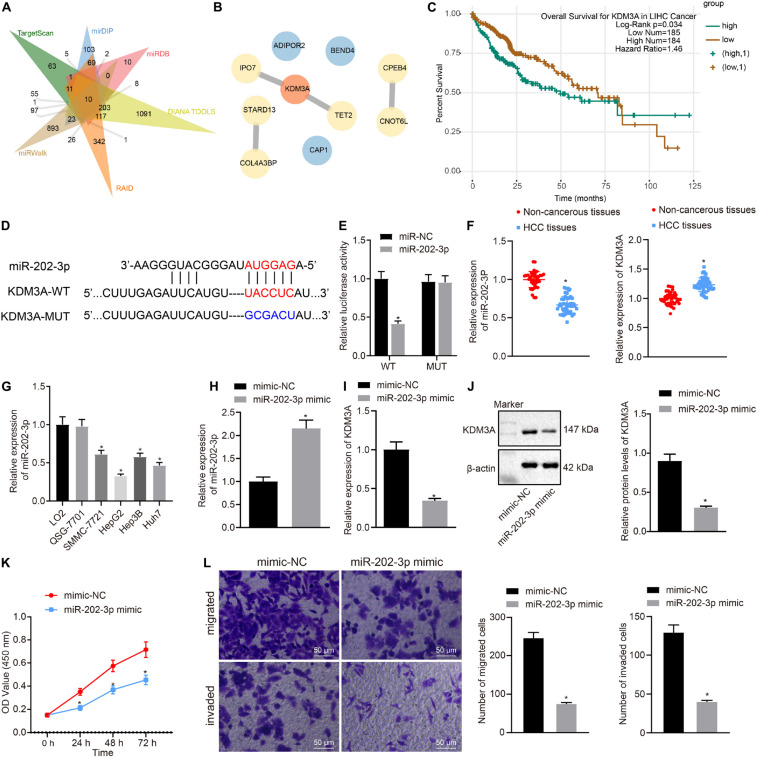
miR-202-3p targets KDM3A gene and inhibits HCC cell viability, migration, and invasion. **(A)** 10 common target genes of miR-202-3p were obtained among the TargetScan (Representative miRNA < –0.25; http://www.targetscan.org/vert_71/), mirDIP (Integrated Score > 0.3; http://ophid.utoronto.ca/mirDIP/), miRDB (Target Score > 80; http://www.mirdb.org/), DIANA TOOLS (miTG score > 0.5; http://diana.imis.athena-innovation.gr/DianaTools), RAID (Score > 0.6; http://www.rna-society.org/raid2/index.html), and miRWalk (bindingp = 1,energy < –20,accessibility < 0.01,au > 0.5; http://mirwalk.umm.uni-heidelberg.de/) databases by Venn diagram. **(B)** PPT network of 10 common target genes of miR-202-3p by the String database analysis, wherein the more red the color of the circle where the gene is located, the higher the degree of its core, while the more blue the color, the lower the core degree. **(C)** Survival curves of HCC patients according to KDM3A expression by the starBase database analysis (HR = 1.46, *p* = 0.034). **(D)** Putative miR-202-3p binding sites in the 3’UTR of KDM3A by the TargetScan database analysis. **(E)** Luciferase activity at the promoter of the reporter gene containing KDM3A-WT and KDM3A-MUT in the presence of miR-202-3p mimic or mimic-NC. * indicates *p* < 0.05 compared with KDM3A-WT + mimic-NC by Tukey’s test-corrected one-way ANOVA. **(F)** The expression of miR-202-3p and KDM3A mRNA was determined by qRT-PCR analysis in HCC tissues (*n* = 50) and tumor-free liver tissues (*n* = 38); * indicates *p* < 0.05 compared with tumor-free liver tissues by paired *t*-test. **(G)** The expression of miR-202-3p was determined by qRT-PCR analysis in cultured HCC cells SMMC7721, HepG2, Hep3B, Huh7, and two normal human liver cell line cell lines LO2 and QSG-7701. * indicates *p* < 0.05 compared with LO2 and QSG-7701 cell lines by Tukey’s test-corrected one-way ANOVA. **(H–J)** The expression of miR-202-3p and KDM3A was determined by qRT-PCR analysis and Western blot analysis in HepG2 cells following miR-202-3p mimic or mimic-NC treatment. * indicates *p* < 0.05 compared with mimic-NC. **(K)** HepG2 cell viability was evaluated by MTT assay at indicated time points. * indicates *p* < 0.05 compared with mimic-NC by Bonferroni-corrected repeated measures ANOVA. **(L)** Representative view (×200) of HepG2 cells migrating from upper transwell chambers without Matrigel into lower ones and statistics of migrating cells; representative view (×200) of HepG2 cells invading from Matrigel-coated upper transwell chambers into lower ones and statistics of invading cells.

To find out target genes of miR-202-3p in HCC, we performed web-available miR-202-3p-mRNA predictions among the TargetScan, mirDIP, miRDB, DIANA TOOLS, RAID, and miRWalk databases, with flagging of 10 common target genes by the Venn diagram (Figure 1A). Next, the 10 target genes were mapped using the String database, where the KDM3A gene was highlighted as the candidate target gene of miR-202-3p by establishing PPT network (Figure 1B). The starBase database analysis showed KDM3A was associated with poor survival of HCC patients (Figure 1C). The TargetScan database showed putative miR-202-3p binding sites in the 3’UTR of KDM3A (Figure 1D), which was further confirmed by reduced luciferase activity at the promoter of the reporter gene containing KDM3A-WT instead of KDM3A-MUT when miR-202-3p mimic was co-transfected (Figure 1E). Next, we quantified the expression of miR-202-3p and KDM3A in 50 HCC tissues and 38 tumor-free liver tissues. The qRT-PCR analysis determined downregulated miR-202-3p and upregulated KDM3A in HCC tissues when comparable to tumor-free liver tissues (Figure 1F). In parallel, the expression of miR-202-3p was also evaluated in cultured HCC cells SMMC7721, HepG2, Hep3B, Huh7, and two normal human liver cell line cell lines LO2 and QSG-7701. As we expected, miR-202-3p exhibited lower expression levels in all HCC cells than in LO2 and QSG-7701 cells (Figure 1G). To further characterize the functional role of miR-202-3p in HCC, we successfully introduced miR-202-3p mimic into HepG2 cells (Figure 1H). The findings revealed that the elevated expression of miR-202-3p by miR-202-3p mimic diminished the expression of KDM3A at the mRNA level (qRT-PCR analysis) and protein level (Western blot analysis) in HepG2 cells (Figures 1I,J), and also retarded HepG2 cell viability, migration, and invasion, demonstrated by MTT assay and transwell chamber systems (Figures 1K,L).

### KDM3A Increased the Expression of HOXA1 by Erasing the H3K9me2 in HCC Cells

**FIGURE 2:**
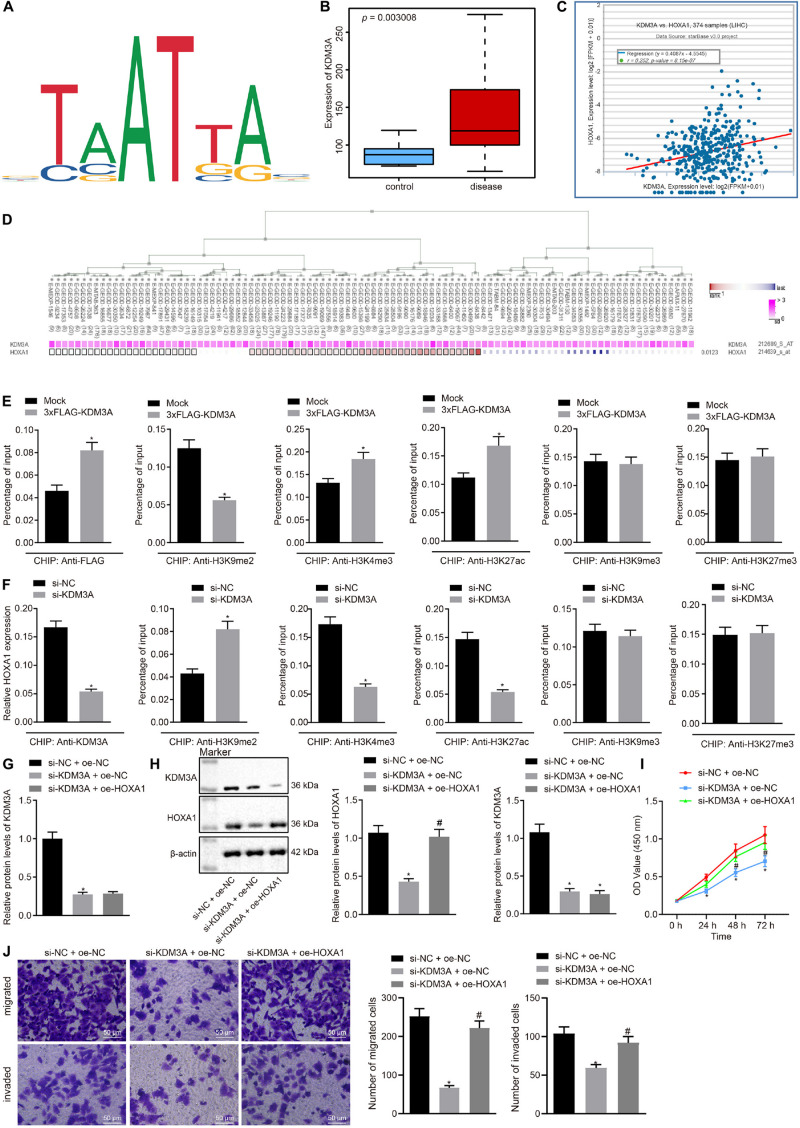
KDM3A binds with the promoter region of HOXA1 gene and increases the expression of HOXA1 by erasing the H3K9me2 in HepG2 cells. **(A)** Methylation-specific binding sites of HOXA1 gene by the JASPAR database (http://jaspar.genereg.net/). **(B)** Expression box of KDM3A between 81 HCC and normal liver tissue specimens after differentially analyzing the GSE62232 dataset (*p* = 3.006E-03). **(C)** Positive correlation between KDM3A and HOXA1 expression by the StarBase database analysis (*r* = 0.252, *p* = 8.15E-07). **(D)** Co-expression of KDM3A and HOXA1 by the MEM analysis (*p* = 0.0123), wherein the abscissa represents different samples; the squares corresponding to the samples and KDM3A represent co-expression intensity of KDM3A and HOXA1; the color of the squares was shown on the upper right, and the deeper the color, the stronger the co-expression intensity of the two; the right side of the squares represents the significance of overall intensity of KDM3A and HOXA1. **(E,F)** ChIP analysis was performed using anti-H3K9me2, anti-H3K9me3, anti-H3K4me3, anti-H3K27ac, and anti-H3K27me3 antibodies in HepG2 cells treated with 3xFLAG-KDM3A or KDM3A siRNA. * indicates *p* < 0.05 compared with Mock or scramble siRNA by paired *t*-test. **(G,H)** Western blots and quantification of KDM3A in HepG2 cells treated with KDM3A siRNA and/or HOXA1 recombinant lentiviral expression vectors. **(I)** HepG2 cell viability was evaluated by MTT assay at indicated time points. **(J)** Representative view (×200) of HepG2 cells migrating from upper transwell chambers without Matrigel into lower ones and statistics of migrating cells; representative view (×200) of HepG2 cells invading from Matrigel-coated upper transwell chambers into lower ones and statistics of invading cells. * (compared with scramble siRNA + oe-NC) and # (compared with KDM3A siRNA + oe-NC) indicate *p* < 0.05 by Tukey’s test-corrected one-way ANOVA for **(G,H,J)** and by Bonferroni-corrected repeated measures ANOVA for **(I)**.

This section of the experiment attempted at characterizing the mechanism of KDM3A in HCC. We obtained methylation-specific binding sites of HOXA1 gene by the JASPAR database (Figure 2A). Then we analyzed differentially expressed genes between 81 HCC and normal liver tissue specimens by using the GSE62232 dataset and found that KDM3A was upregulated in HCC tissue specimens (Figure 2B). The StarBase database analysis identified a positive correlation between KDM3A and HOXA1 expression (Figure 2C), which was further signified by the MEM analysis (Figure 2D).

To test whether KDM3A regulates HOXA1 expression at the transcriptional level, ChIP analysis was performed using anti-H3K9me2, anti-H3K9me3, anti-H3K4me3, anti-H3K27ac, anti-H3K27me3 antibodies in HepG2 cells treated with KDM3A siRNA. We found that KDM3A protein was highly enriched in the promoter region of HOXA1 gene in HepG2 cells following 3xFLAG-KDM3A transfection. KDM3A siRNA treatment significantly amplified H3K9me2 enrichment but reduced H3K4me3 and H3K27ac enrichment in the promoter region of HOXA1 gene (Figures 2E,F). No significant difference was evident regarding H3K9me3 and H3K27me3 enrichment in the promoter region of HOXA1 gene. These data suggest that KDM3A could bind with the promoter region of HOXA1 gene and increase the expression of HOXA1 by erasing the H3K9me2 in HepG2 cells. To further verify the regulation of KDM3A on HOXA1, we manipulated the expression of KDM3A and HOXA1 in HepG2 cells using KDM3A siRNA and HOXA1 recombinant lentiviral expression vectors. The results (Figures 2G–J) of Western blot analysis, MTT assay, and transwell chamber systems found that KDM3A siRNA treatment reduced the expression of HOXA1 and inhibited HepG2 cell viability, migration, and invasion. Predictably, our findings highlighted that the HOXA1 overexpression restored the viability, migration, and invasion of KDM3A knockdown HepG2 cells.

### HOXA1 Promoted HCC Cell Viability, Migration, and Invasion by Binding With the MEIS3 Enhancer Region

**FIGURE 3:**
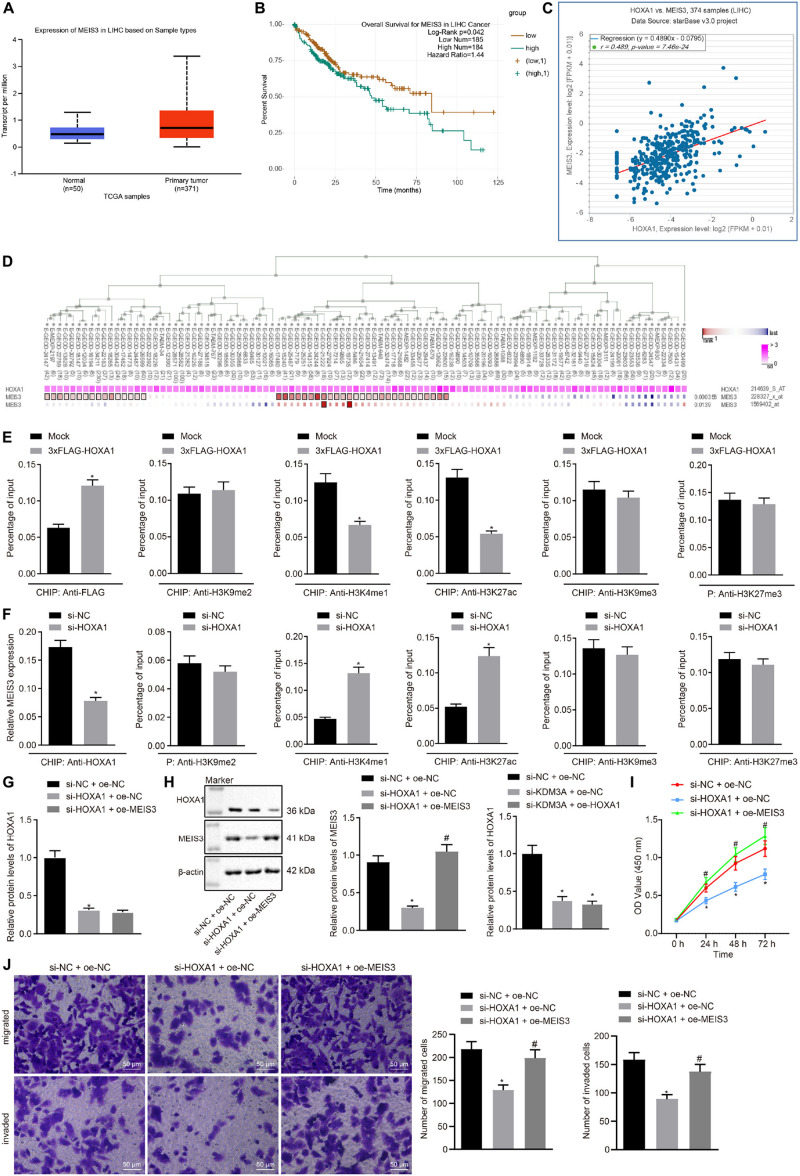
HOXA1 promotes HCC cell viability, migration, and invasion by binding with the MEIS3 enhancer region. **(A)** Expression box of MEIS3 in HCC by the Ualcan database analysis. **(B)** Survival curves of HCC patients according to MEIS3 expression by the starBase database analysis (HR = 1.44, *p* = 0.042). **(C)** Positive correlation between HOXA1 and MEIS3 expression by the StarBase database analysis (*r* = 0.489, *p* = 7.46E-24). **(D)** Co-expression of HOXA1 and MEIS3 by the MEM analysis (*p* = 3.56E-04), wherein the abscissa represents different samples; the squares corresponding to the samples and KDM3A represent co-expression intensity of MEIS3 and HOXA1; the color of the squares was shown on the upper right, and the deeper the color, the stronger the co-expression intensity of the two; the right side of the squares represents the significance of overall intensity of MEIS3 and HOXA1. **(E,F)** ChIP analysis was performed using anti-H3K9me2, anti-H3K4me1, anti-H3K27ac, anti-H3K9me3, and anti-H3K27me3 antibodies in HepG2 cells treated with 3xFLAG-HOXA1 or HOXA1 siRNA. * indicates *p* < 0.05 compared with Mock or scramble siRNA by paired *t*-test. **(G,H)** Western blots and quantification of MEIS3 in HepG2 cells treated with HOXA1 siRNA and/or MEIS3 recombinant lentiviral expression vectors. **(I)** HepG2 cell viability was evaluated by MTT assay at indicated time points. **(J)** Representative view (×200) of HepG2 cells migrating from upper transwell chambers without Matrigel into lower ones and statistics of migrating cells; representative view (×200) of HepG2 cells invading from Matrigel-coated upper transwell chambers into lower ones and statistics of invading cells. * (compared with scramble siRNA + oe-NC) and # (compared with HOXA1 siRNA + oe-NC) indicate *p* < 0.05 by Tukey’s test-corrected one-way ANOVA for **(G,H,J)** and by Bonferroni-corrected repeated measures ANOVA for **(I)**.

The ability of HOXA1 to bind to the MEIS3 enhancer region has been elucidated by preceding evidence (De Kumar et al., 2017). On this basis, it was hypothesized that HOXA1 is involved in the development of HCC by binding with the MEIS3 enhancer region. The Ualcan database analysis showed MEIS3 was upregulated in HCC (Figure 3A). The starBase database analysis elicited the association between MEIS3 with poor survival of HCC patients (Figure 3B), and further revealed the presence of a positive correlation between HOXA1 and MEIS3 expression (Figure 3C), which was further signified by the MEM analysis (Figure 3D).

To test whether HOXA1 regulates MEIS3 expression at the transcriptional level, ChIP analysis was performed using anti-H3K9me2, anti-H3K4me1, anti-H3K27ac, anti-H3K9me3, and anti-H3K27me3 antibodies in HepG2 cells treated with MEIS3 siRNA. Our data revealed that HOXA1 protein was highly enriched in the MEIS3 enhancer region in HepG2 cells following 3xFLAG-HOXA1 transfection. Similarly, H3K4me1 and H3K27ac enrichment was evident in comparison to H3K9me2, H3K9me3, and H3K27me3 in the MEIS3 enhancer region in HepG2 cells. Expectedly, HOXA1 siRNA treatment significantly reduced H3K4me1 and H3K27ac enrichment in the MEIS3 enhancer region (Figures 3E,F), with no impact on H3K9me2, H3K9me3, and H3K27me3 enrichment. These data suggest that HOXA1 could bind with the MEIS3 enhancer region and increase the expression of MEIS3 by enhancing H3K4me1 and H3K27ac modification in HepG2 cells. In an attempt to further verify the regulation of HOXA1 on MEIS3, we manipulated the expression of HOXA1 and MEIS3 in HepG2 cells using HOXA1 siRNA and MEIS3 recombinant lentiviral expression vectors. The results (Figures 3G–J) of Western blot analysis, MTT assay, and transwell chamber systems found that HOXA1 siRNA treatment reduced the expression of MEIS3 and inhibited HepG2 cell viability, migration, and invasion. Expectedly, MEIS3 overexpression was found to restore the viability, migration, and invasion of HOXA1 knockdown HepG2 cells.

### miR-202-3p Suppressed HCC Cells *in vitro* by the KDM3A/HOXA1/MEIS3 Pathway

**FIGURE 4:**
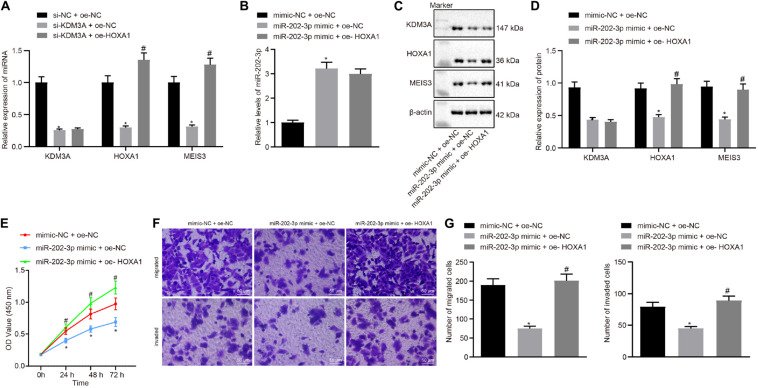
miR-202-3p suppresses HepG2 cell viability, migration and invasion *in vitro* by the KDM3A/HOXA1/MEIS3 pathway. **(A)** The mRNA level of KDM3A, HOXA1, and MESI3 in KDM3A knockdown and/or HOXA1 overexpression HepG2 cells by qRT-PCR analysis. * (compared with scramble siRNA + oe-NC) and # (compared with KDM3A siRNA + oe-NC) indicate *p* < 0.05 by Tukey’s test-corrected one-way ANOVA. **(B–D)** The miR-202-3p expression, Western blots, and quantification of KDM3A, HOXA1, and MESI3 in HepG2 cells treated with miR-202-3p mimic alone or with HOXA1 recombinant lentiviral expression vectors by qRT-PCR and Western blot analysis. **(E)** HepG2 cell viability was evaluated by MTT assay at indicated time points. **(F,G)** Representative view (×200) of HepG2 cells migrating from upper transwell chambers without Matrigel into lower ones and statistics of migrating cells; representative view (×200) of HepG2 cells invading from Matrigel-coated upper transwell chambers into lower ones and statistics of invading cells. * (compared with mimic-NC + oe-NC) and # (compared with miR-202-3p mimic + oe-NC) indicate *p* < 0.05 by Tukey’s test-corrected one-way ANOVA for **(B–D)** and **(F,G)** and by Bonferroni-corrected repeated measures ANOVA for **(E)**.

In view of these findings, our next endeavor is to verify the involvement of the miR-202-3p/KDM3A/HOXA1/MEIS3 pathway in HCC cells. We examined the mRNA level of KDM3A, HOXA1, and MESI3 in KDM3A knockdown and/or HOXA1 overexpression HepG2 cells. The results of qRT-PCR analysis found that KDM3A knockdown reduced the mRNA level of KDM3A, HOXA1, and MESI3 in HepG2 cells, while HOXA1 overexpression restored the mRNA level of MESI3 against KDM3A knockdown in HepG2 cells (Figure 4A). To further identify miR-202-3p as a functional regulator of the KDM3A/HOXA1/MEIS3 pathway in HCC, HepG2 cells were treated with miR-202-3p mimic alone or a combination of HOXA1 recombinant lentiviral expression vectors. The results of qRT-PCR and Western blot analysis displayed that elevated expression of miR-202-3p by its specific mimic diminished the protein expression of KDM3A, HOXA1, and MEIS3 in HepG2 cells (Figures 4B–D). Likewise, HOXA1 overexpression restored the protein expression of HOXA1 and MEIS3 against miR-202-3p mimic in HepG2 cells. Next, we performed MTT assay and transwell chamber systems, and found that HOXA1 overexpression rescued HepG2 cell viability, migration, and invasion from miR-202-3p mimic (Figures 4E–G).

### miR-202-3p Suppressed the *in vivo* Tumorigenicity of HCC Cells by the KDM3A/HOXA1/MEIS3 Pathway

**FIGURE 5:**
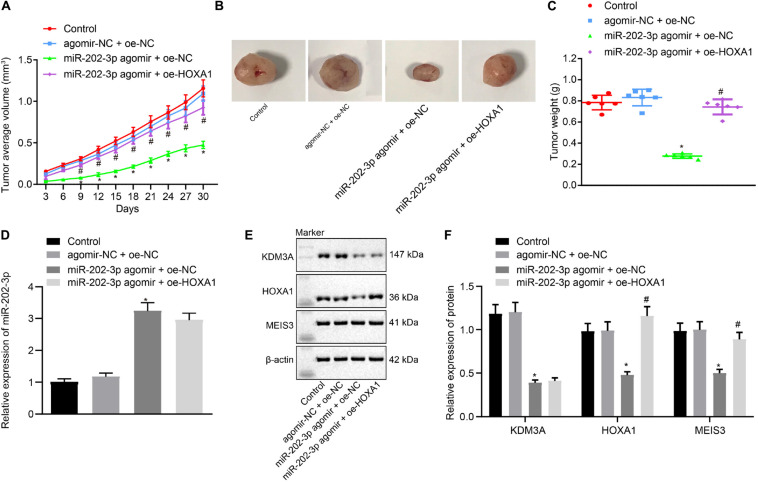
miR-202-3p inhibits the *in vivo* tumorigenicity of HCC cells by the KDM3A/HOXA1/MEIS3 pathway. **(A)** Tumor volume of HepG2 cells xenografted nude mice at indicated time points. **(B)** Representative mouse xenotransplanted tumors of HepG2 cells. **(C)** The scatter diagram of tumor weight of HepG2 cells xenografted nude mice. **(D)** The expression of miR-202-3p in tumor tissues collected from HepG2 cells xenografted nude mice was determined by qRT-PCR analysis. **(E,F)** Western blots and quantification of KDM3A, HOXA1, and MESI3 in tumor tissues collected from HepG2 cells xenografted nude mice. * (compared with control or agomir-NC + oe-NC) and # (compared with miR-202-3p agomir + oe-NC) indicate *p* < 0.05 by Tukey’s test-corrected one-way ANOVA for **(C–F)** and by Bonferroni-corrected repeated measures ANOVA for **(A)**.

To verify the findings of *in vitro* experiments, nude mice were subcutaneously injected with miR-202-3p agomir. Our data revealed that miR-202-3p elevation suppressed the tumor growth of HepG2 cell xenografted into nude mice, as reflected by reduced tumor weight and volume in nude mice injected with miR-202-3p, which the addition of oe-HOXA1 could reverse this effect (Figures 5A–C). Mouse tumor tissues were subject to qRT-PCR and Western blot analysis, and the results demonstrated that the miR-202-3p agomir diminished the protein expression of KDM3A, HOXA1, and MEIS3 in nude mice. Then, HepG2 cells with lentivirus-mediated HOXA1 overexpression were xenografted into nude mice, and the mice were injected with miR-202-3p agomir. The data showed that lentivirus-mediated HOXA1 overexpression restored the protein expression of HOXA1 and MEIS3 against miR-202-3p agomir in nude mice (Figures 5D–F).

## Discussion

Statistics show that liver cancer ranks the seventh prevalently occurring malignancy and is considered as a leading cause of death related to cancers, accounting for 4.7 and 8.2%, respectively, in both sexes on a global scale as reported in 2018 (Bray et al., 2018). Nowadays, the focus of research has shifted to elucidating the significance of various miRNAs in liver tumorigenesis from perspectives of diagnosis, treatment modalities, and oncologic outcomes (Drakaki et al., 2013; Gailhouste et al., 2013; Callegari et al., 2015). Our current investigation aimed at exploring the functionality role of miR-202-3p in HCC through its interplay with its downstream regulators. Collectively, the experimental data demonstrated that miR-202-3p harbored tumor-suppressive properties during the development and progression of HCC through the KDM3A/HOXA1/MEIS3 axis *in vitro* and *in vivo*.

A fundamental finding of our study was the low expression of miR-202-3p and high expression of KDM3A in clinically collected HCC tissues and commercially purchased cell lines. A study based on colorectal cancer documented similar findings in terms of miR-202-3p expression pattern supporting that miR-202-3p is poorly expressed in colorectal cancer samples and upregulated miR-202-3p exerts tumor-inhibiting activities (Wang et al., 2014). Likewise, miR-202-3p has been indicated to be downregulated in cervical cancer and papillary thyroid carcinoma with implication in modulation of malignant cellular behaviors (Chen et al., 2019; Han et al., 2019). In addition, miR-202-3p inhibited gastric cancer activity by inhibiting the expression of catenin and BCL-2 (Zhao et al., 2013). miR-202-3p suppressed the WNT signaling pathway by downregulating the expression of catenin in TPC-1 and BCPAP cells (Chen et al., 2019). miR-202-3p can also retard tumors by downregulating adp-ribosylation factor-like 5A protein level in colorectal cancer (Wang et al., 2014). Of note, histone demethylase KDM3A, also termed as JMJD1A, has been found to be upregulated in bladder cancer and lung adenocarcinoma while knockdown of KDM3A possesses therapeutic potential (Cho et al., 2012; Li et al., 2017). Upregulated KDM3A in HCC has been proposed as a biomarker of recurrence following hepatic resection in association with malignant transformation (Yamada et al., 2012). Notably, an existing study validated the ability of overexpressed miR-202 to exercise inhibitory effects on proliferative capacity and tumorigenicity of HCC cells (Zhang et al., 2014). Concordantly, subsequent experiments were performed in our study for exploration purpose by delivering miR-202-3p mimic or siRNA targeting KDM3A, leading to suppressed proliferation, migration, and invasion capabilities of HCC cells *in vitro* as well as weakened tumorigenicity *in vivo*.

Further investigation regarding the molecular mechanism revealed that KDM3A was targeted and negatively regulated by miR-202-3p and HOXA1 was required for the regulatory role of KDM3A in HCC. As a recognized H3K9 demethylase, KDM3A has been identified to be pro-tumorigenic in hepatotumorigenesis (Nakatsuka et al., 2017). The depletion of KDM3A has also been observed to cause inhibited tumorigenicity of HCC cells under hypoxic condition both *in vitro* and *in vivo* (Park et al., 2013). It has been reported that KDM3A can promote the expression of HOXA1 in a direct manner by binding to the promoter region of HOXA1 through demethylation of H3K9me2 (Cho et al., 2012). Also, activity of histone demethylase KDM3A has been found to play an important part in the mediation of oncogene HOXA1 expression in breast cancer (Mahajan et al., 2014). Furthermore, reports have elucidated the upregulation of HOXA1 in close association with the dismal oncologic outcomes of patients with HCC while downregulation of HOXA1 corresponds to suppressed growth, migration, and invasion of HCC cells (Zha et al., 2012). Consistently, our results demonstrated that the presence of overexpressed HOXA1 could override the depleted KDM3A-induced inhibitory effects on HCC cell proliferation, migration, and invasion *in vitro* as well as tumorigenic ability *in vivo*. Endogenous HOXA1 protein was further demonstrated to bind to the MEIS3 enhancer to promote its expression by activating enhancer through modification of histone H3K4me1 and H3K27ac. Concordantly, there is available evidence revealing MEIS3 as a downstream target of HOXA1 that HOXA1 can bind to MEIS3 enhancer to elevate its expression level (De Kumar et al., 2017). In addition, MEIS3, a member of the MEIS family involved in vertebrate tumorigenesis, has been detected to be expressed at a low level in ovarian carcinoma cells, hereby validating its functionality in regulating malignant cell survival (Liu et al., 2010). Nevertheless, after the treatment of overexpressed MEIS3, the suppressive effects triggered by HOXA1 knockdown were compensated, which warrants for the need of further studies based on the expression profile determination of MEIS3 in HCC.

**FIGURE 6:**
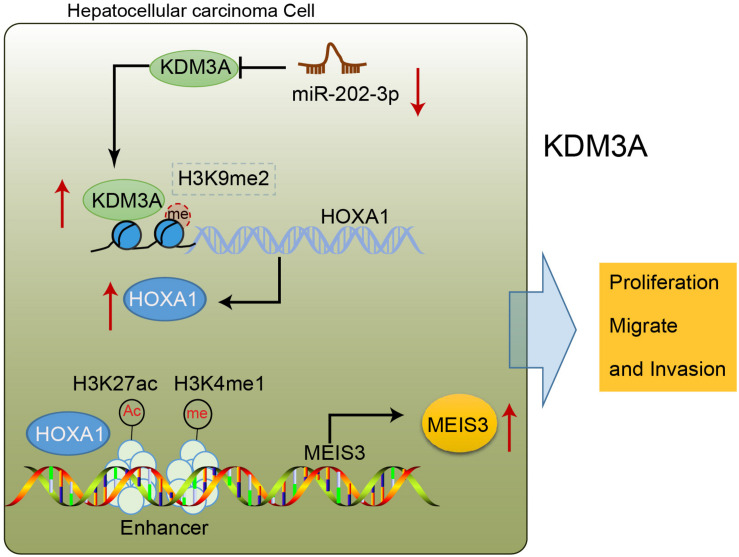
miR-202-3p targets KDM3A, and the demethylase KDM3A binds to the promoter region of HOXA1, removes H3K9me2 modification, and enhances the expression of HOXA1; HOXA1 binds to the enhancer of MEIS3, enriches H3K4me1 and H3K27ac modification in the enhancer region, and activates the enhancer to promote the expression of MEIS3, thereby promoting the proliferation, migration, and invasion of HCC cells.

To sum up, our findings shed new light on the tumor-suppressive action of miR-202-3p, underscoring the therapeutic significance of a putative candidate against development and progression of HCC (Figure 6). Furthermore, the downstream axis involving KDM3A/HOXA1/MEIS3 was proposed to be essential for miR-202-3p to facilitate its functionality as a tumor suppressor. However, further studies are still warranted for future clinical application deliberating that the development of molecular targeted therapies is still in its infancy and has not been materialized.

## Data Availability Statement

The original contributions presented in the study are included in the article/supplementary material. Further inquiries can be directed to the corresponding author/s.

## Ethics Statement

The studies involving human participants were reviewed and approved by the First Affiliated Hospital of China Medical University. The patients/participants provided their written informed consent to participate in this study. The animal study was reviewed and approved by the First Affiliated Hospital of China Medical University.

## Author Contributions

YZ, QP, and ZS participated in the design of the experiments, analyzed and interpreted the data, and assembled the figures. YZ and ZS formulated the study concept and all experimental designs, supervised the project, and wrote the manuscript. All authors contributed to the article and approved the submitted version.

## Conflict of Interest

The authors declare that the research was conducted in the absence of any commercial or financial relationships that could be construed as a potential conflict of interest.

## Footnotes

1 http://www.targetscan.org/vert_71/

2 http://ophid.utoronto.ca/mirDIP/

3 http://www.mirdb.org/

4 http://diana.imis.athena-innovation.gr/DianaTools

5 http://www.rna-society.org/raid2/index.html

6 http://mirwalk.umm.uni-heidelberg.de/

7 https://string-db.org/

8 https://cytoscape.org/

9 https://www.ncbi.nlm.nih.gov/gds

10 http://jaspar.genereg.net/

11 http://starbase.sysu.edu.cn/

12 https://biit.cs.ut.ee/mem/index.cgi

13 http://ualcan.path.uab.edu/index.html

## Abbreviations

ANOVA: analysis of variance
ChIP: chromatin immunoprecipitation
DMSO: dimethyl sulfoxide
ECL: chemiluminescence
H3K9: histone H3 lysine 9
HCC: hepatocellular carcinoma
HOXA1: homeobox A1
FBS: fetal bovine serum
miR-202-3p: microRNA-202-3p
PAGE: polyacrylamide gel electrophoresis
PDK1: protein kinase 1
PMSF: phenylmethylsulfonyl fluoride
RIPA: radioimmunoprecipitation
qRT-PCR: quantitative real-time PCR
SDS: sodium dodecyl sulfate
SPF: specific pathogen-free.
